# Prevalence of *Coxiella burnetii* seropositivity and shedding in farm, pet and feral cats and associated risk factors in farm cats in Quebec, Canada

**DOI:** 10.1017/S0950268821000364

**Published:** 2021-02-15

**Authors:** J. Cyr, M.-È. Turcotte, A. Desrosiers, D. Bélanger, J. Harel, D. Tremblay, A. Leboeuf, C. A. Gagnon, J.-C. Côté, J. Arsenault

**Affiliations:** 1 Faculté de médecine vétérinaire, Université de Montréal, Saint-Hyacinthe, QC, Canada; 2 Groupe de Recherche en Épidémiologie des Zoonoses et Santé Publique (GREZOSP), Université de Montréal, Saint-Hyacinthe, QC, Canada; 3 Faculté de médecine vétérinaire, Université de Montréal, Swine and poultry infectious diseases research center (CRIPA) – Fonds de Recherche du Québec, Saint-Hyacinthe, QC, Canada; 4 Service de diagnostic, Faculté de médecine vétérinaire, Université de Montréal, Saint-Hyacinthe, QC, Canada; 5 Ministère de l'Agriculture, des Pêcheries et de l'Alimentation du Québec, Quebec, QC, Canada

**Keywords:** cats, *Coxiella burnetii*, prevalence, risk factors

## Abstract

Cats represent a potential source of *Coxiella burnetii*, the aetiological agent of Q fever in humans. The prevalence and risk factors of *C. burnetii* infection in farm, pet and feral cats were studied in Quebec, Canada, using a cross-sectional study. Serum samples were tested using a specific enzyme-linked immunosorbent assay (ELISA) for the presence of antibodies against *C. burnetii*, whereas rectal swabs were assayed using real-time quantitative polymerase chain reaction (qPCR) for the molecular detection of the bacteria. Potential risk factors for farm cats were investigated using clinical examinations, questionnaires and results from a concurrent study on *C. burnetii* farm status. A total of 184 cats were tested: 59 from ruminant farms, 73 pets and 52 feral cats. Among farm cats, 2/59 (3.4%) were ELISA-positive, 3/59 (5.1%) were ELISA-doubtful and 1/59 (1.7%) was qPCR-positive. All pets and feral cats were negative to *C. burnetii* ELISA and qPCR. Farm cat positivity was associated with a positive *C. burnetii* status on the ruminant farm (prevalence ratio = 7.6, *P* = 0.03). Our results suggest that although pet and feral cats do not seem to pose a great *C. burnetii* risk to public health, more active care should be taken when in contact with cats from ruminant farms.

## Introduction

Q fever is a zoonotic disease caused by *Coxiella burnetii*, an obligate intracellular, Gram-negative bacterium [1]. Human Q fever has been documented in many parts of the world [2]. The bacteria can infect a wide range of hosts, including domestic cattle, sheep and goats, companion animals, cats and dogs and several vertebrate and invertebrate wildlife species [1, 3, 4]. Experimental findings have shown that a single bacterium could initiate infection in humans [5]. Although most human infection cases remain asymptomatic or develop flu-like symptoms, fever, myalgia and/or headache, serious acute Q fever may lead to abortion, pneumonia, hepatitis, pericarditis, myocarditis, endocarditis and meningitis, and may further develop into persistent infection [1].

The most common mode of transmission to humans is airborne by inhalation of aerosol particles contaminated with parturient secretions from infected animals [6]. *Coxiella burnetii* is resistant to drying and can survive in the soil for several weeks [7]. Although human infections have historically been mostly associated with close contact with domestic ruminants [1, 8], cats that live in close proximity to humans are regarded as a potential source of *C. burnetii* [9–11]. Many studies reported the detection of antibodies against phase I and/or phase II *C. burnetii* antigens in cat sera [12–15], and some cases of human Q fever were linked with exposure to parturient cats [16–19]. Although little is known on the main sources of infection in cats, it has been proposed that cats may become infected by consumption of placenta or milk from infected ruminants, consumption of contaminated raw meat intended for pet consumption, inhalation from environmental contamination, ingestion of infected prey, or tick bites [3, 20, 21]. Considering evidence supporting that rats could be involved as reservoirs for *C. burnetii*, they could maintain the bacterial infection in preying cats [22]. Some studies reported inconsistent differences in the prevalence of *C. burnetii* infection between client-owned and shelter or stray cats [9, 10, 12]. To the best of our knowledge, no study has been conducted to evaluate the risk of *C. burnetii* infection in farm cats, which may be more likely to be exposed owing to their close contact with domestic ruminants. In addition, although some studies confirmed the presence of *C. burnetii* in the reproductive system of female cats [10, 11], no study that we know of has used PCR to screen cat faeces for the presence and shedding of the bacteria.

The purpose of this study was two-fold: (1) to estimate the prevalence of *C. burnetii* seropositivity and faecal shedding in cats living on farms, in pet cats from households and in feral cats from an urban environment, and (2) to identify risk factors associated with seropositivity and/or shedding of *C. burnetii*.

## Methods

### Study design and cat selection

A cross-sectional study was performed in 2011 in three regions (Rimouski-Neigette/La Mitis, Les Maskoutains, Montreal) encompassing four regional county municipalities (MRC), which are administrative areas used in Quebec, Canada (Supplementary Fig. S1). The first two regions included the MRC of Rimouski-Neigette and Les Maskoutains, respectively. They were mostly rural and were selected for the farm and pet cat samplings as part of a concurrent study on *C. burnetii* in ruminants [23]. Besides, another MRC adjacent to Rimouski-Neigette, ‘La Mitis’, was added for pet cat sampling in this region. The MRC of Montreal, which is the largest urban area in Quebec, Canada, was also included for pet cat sampling and to investigate whether feral cats could represent a source of *C. burnetii* infection in a densely populated city.

The selection of cats was based on three distinct categories: farm cats, pet cats and feral cats. The target sample size was calculated to include at least 60 cats per category from a combination of all regions. This number was estimated using an online calculator [24] to allow the detection of at least one *C. burnetii*-enzyme-linked immunosorbent assay (ELISA)- or -quantitative polymerase chain reaction (qPCR)-positive cat with a confidence level of 95%, assuming a prevalence of at least 5% in each cat category. This approach was chosen given (1) the absence of prior information on the expected apparent prevalence in this specific population, (2) the absence of prior information on the diagnostic performance of the assays in regards to the targeted species, (3) the consideration that from a public health risk, a prevalence of at least 5% was considered worrisome and would need to be reported, (4) a sample size of 60 per group was sufficient to estimate an apparent prevalence of 4% with a 5% error, or an estimated apparent prevalence of 20% with a precision of 10%.

Farm cats were defined as cats that live on dairy cow, sheep or goat farms. Cats were selected from a total of 107 farms located in the Rimouski-Neigette or Les Maskoutains. These farms were initially recruited for a concurrent study on the prevalence and risk factors of *C. burnetii* infection in farm animals [23] from a random selection of agricultural enterprises registered with the Quebec Ministry of Agriculture, Fisheries and Food, Canada and having at least 15 breeding females. All dairy cattle farms operate in all-year-round calving system, whereas various breeding management systems were used in small ruminant farms. Ninety-two farms had at least one cat on the farm and were asked to participate in the study on a voluntary basis. In cases where more than one cat was present on a farm, a single one was selected by convenience by the research team. Farm cats were sampled on the site with the farmer's help. No financial compensation was provided to the farmer.

Pet cats were defined as indoor or at least partially indoor owned cats. They were gathered at veterinary clinics from all regions. From the list of all pet veterinary clinics located in these areas, clinics were randomly selected and invited to participate in our study until two clinics by region were recruited. Each clinic was asked to recruit a target of 10 cats. Cats were chosen at each clinic by their respective staff following specific inclusion criteria: (1) the cat was 12 weeks of age or older, (2) the owner lived in the MRC as the clinic location, (3) the owner was fluent in French and (4) the owner provided informed written consent for participation. At the beginning of the study, two additional criteria were used: (1) the cat belonged to the same owner since 2 months of age or younger, and (2) the owner did not move to a new dwelling since owning the cat. However, due to difficulties in recruiting cats meeting all inclusion criteria, these two criteria were discarded as they were not expected to be associated with cat exposure status, but were rather initially used to allow for a lifelong appraisal of the potential risk factors, which was then revised to only consider exposures over the last 6 months. Sampling was extended to the summer of 2012 to reach the sample size. Only one cat per client was included in the study. A financial compensation of 20$CAN per sampled cat was provided to the veterinary clinics.

All feral cats were taken from the Montreal region. They were street cats living in outdoor colonies and caught within the ‘Trap-Neuter-Release-Maintain Program’ of the Montreal division of the Society for the Prevention of Cruelty to Animals (SPCA). This programme consists of trapping, sterilising, vaccination and deworming stray and feral cats and returning them to their colony. Cats were caught using TRU-CATCH^©^ traps (Belle Fourche, SD, USA) with food bait under the surveillance of volunteers. No financial compensation was given to the SPCA, except that SNAP Feline Triple Tests (IDEXX, Westbrook, ME, USA) were provided to their veterinarian for screening cats for feline immunodeficiency virus (FIV), feline leukaemia virus (FeLV) and feline heartworm infection.

### Cat sampling and physical examination

All farm cat sampling was performed by the research team. Farm cat blood samples were taken using a 1 ml Terumo® syringe (Fisher Scientific, Ottawa, ON, Canada) with a size 25 G × 5/8″ needle (Fisher Scientific) in the femoral vein. When the femoral vein sampling failed, blood was collected from the jugular vein. Between 0.5 and 1 ml of blood was sampled for each cat. After needle removal, the pressure was placed on the puncture site for 1–2 min to prevent haematoma. Blood was immediately transferred in a Monoject™ blood collection tube (VWR, Mississauga, ON, Canada) with no additive. Rectal swabbing was performed with a Copan® 150C cotton swab in tubes pre-wetted with sterile water. Blood samples and rectal swabs were put on ice and transferred to the laboratory for analyses within 24–32 h. Before release, a physical exam was conducted to determine the sex, estimate the age group (<6 months, 6 months to 1 year, >1 year) and assess the presence of suggestive signs of sterilisation (i.e. spay scar in female cats), gestation/lactation and any clinical signs suggestive of a disease. The body condition was scored on a four-level scale (overweight, normal, thin or emaciated).

Pet cat blood sampling and rectal swabbing were done by the staff at each veterinary clinic using their own routine method for blood taking (the jugular vein for cooperative cats and the femoral vein for more agitated cats). Monoject™ blood collection tubes with no additive and Copan^®^ 150C cotton swabs in tubes were supplied to the clinics for the exclusive purpose of this study. Following sampling, a physical exam was conducted as described above by a veterinarian of the clinic and included the reason for consultation.

Feral cat blood sampling and rectal swab were done by the staff of the SPCA clinic following their preferred method for drawing blood on cats. Monoject™ blood collection tubes with no additive and Copan^®^ 150C cotton swabs in tubes were supplied to the SPCA. A physical exam was conducted as described above by the SPCA staff. In addition, the district of capture and status for FIV and FeLV was determined using the SNAP Feline Triple Test.

### Questionnaires

Two questionnaires were developed for the evaluation of potential risk factors, one for farm cats and another for pet cats. Information was gathered on age, sex, sterilisation, prior litters and history of abortion, the origin of the cat, and contact history with potential sources of the bacteria. Both questionnaires were filled out by the research team during a face-to-face interview with the cat owner at the time of sampling (for farm cats only) or during a phone interview in the 2 weeks following sampling (for farm and pet cats). The cat owner and the interviewer were blinded to *C. burnetii* status determined during the study for the cat and the farm of origin. Both questionnaires were written in French and are available upon request.

### Determination of farm status

For farm cats, the *C. burnetii* status of livestock was obtained from our concurrent study [23]. Briefly, for dairy cattle, the status of the farm was determined by ELISA and qPCR assays of three bulk tank milk samples collected 3 to 5 weeks apart. For small ruminants, sera and faeces from 15 females, aged 6 months and older, and born on the farm were tested by ELISA and qPCR, respectively. Both tests were conducted as described below for cats [23]. A farm was considered *C. burnetii*-ELISA-positive or *C. burnetii*-ELISA-doubtful when at least one sample was positive or doubtful, respectively, and as *C. burnetii-*qPCR positive when at least one sample was qPCR-positive.

### Enzyme-linked immunosorbent assay for detection of antibodies to *C. burnetii*

Serum samples were tested using the ID Screen® Q Fever Indirect Multi-species ELISA kit (IDVet, Grabels, France), which detects anti-*C. burnetii* antibodies to the naturally occurring phases I and II variant. The ELISA results were determined using the ELx808^TM^ absorbance microplate reader (BioTek, Winooski, VT, United States). Sample to positive control (S/P) ratio (%) was used for test interpretation, with S/P < 40% considered as negative, between 40% and 50% inclusively as doubtful, and greater than 50% as positive, as recommended by the manufacturer.

### Real-time quantitative polymerase chain reaction for detection of *C. burnetii*

Rectal swabs were suspended in 0.5 ml of PBS buffer, 200 μl of the suspension were used for DNA extraction with the QIAamp DNA Mini Kit following the manufacturer's recommendations (Qiagen, Toronto, ON, Canada) and eluted in 50 μl of AE buffer (10 mM Tris-Cl, 0.5 mM EDTA; pH 9.0). A volume of 5 μl of the template was used in the qPCR reaction using primers and probe for the amplification and detection of the *icd* (Isocitrate dehydrogenase [NADP]) gene as described by [25] except that the Black Hole Quencher (Biosearch Technologies, Petaluma, CA, USA) was used instead of the TAMRA Quencher for the probe. Black Hole Quencher dyes are highly efficient with no native florescence emission, which simplifies the interpretation of qPCR assays. Positive (*C. burnetii* genomic DNA) and negative controls were included in each run. Samples showing *Cq* (threshold cycle) values <40 were considered positive. Using a calibration curve made with a known quantity of the *icd* gene copies, the *Cq* values of positive samples were used to extrapolate *C. burnetii* genomic copy number within the tested samples. As previously shown for a similar PCR test, this assay has high analytic sensitivity and specificity [25].

### Data analysis

Descriptive statistics were used to present the results. Statistical analyses were performed separately for the three cat categories (farm, pet and feral) given differences in sampling methods and sets of risk factors investigated. For each category, the prevalence of *C. burnetii*-ELISA-positive, *C. burnetii*-ELISA-doubtful and-qPCR positive cats with a 95% confidence interval (CI) was estimated based on the exact binomial distribution. The risk factor analyses were limited to farm cats due to the absence of positive in pet and feral cats. The potential risk factors associated with bacterial shedding (i.e. qPCR-positive) and with a previous infection of the cat were evaluated using Fisher's exact test. Two case definitions of the cat's previous infection status were used to assess associations: a first one in which the cat was ELISA-positive and/or qPCR-positive, and a second one, in which the cat was ELISA-doubtful, ELISA-positive and/or qPCR-positive. Based on the literature, risk factors directly related to exposure to a previously suspected or reported source of *C.burnetii*, or related to the expression of clinical signs suggestive of a past infection, were assessed using one-sided tests (i.e. exposure or expression of clinical signs was expected to increase the risk of cat positivity); otherwise, exploratory two-sided tests were performed. Prevalence ratios (PR) were used to present statistically significant associations, with the *alpha* value set at 0.05. No multivariable analyses were performed due to the low number of positives and limited sample size. All analyses were performed in SAS version 9.4 software (SAS, Cary, NC, USA).

## Results

A total of 184 cats were included in this study. They included 59 farm, 73 pet- and 52 feral cats. The distribution of the farm-, pet- and feral cats in each region studied is illustrated in Supplementary Fig. S1.

All but 1 of the 92 producers with at least 1 cat on the farm agreed to participate in the study. A total of 59 farm cats were sampled between 1 June 2011 and 6 July 2011 from two regions, Rimouski-Neigette and Les Maskoutains (Table 1). Most cats were cooperative and could be picked directly. Some cats had to be baited with food. In rare instances, upon advance notice, some farmers trapped their cats in an isolated room earlier in the day. The absence of cat sampling was mostly due to the inability to find any cat on the farm at the time of the visit.

**Table 1.** Characteristics of cats and associated *Coxiella burnetii* status in farm cats sampled in two regions of Quebec, Canada, in 2011

Characteristic	qPCR		qPCR and ELISA		
	Number of farm cats (*n* = 59)	Number (%) of positive	Number of farm cats (*n* = 59)	Number (%) of positive^a^	Number (%) of doubtful or positive^b^
Region					
Rimouski-Neigette	24	0	24	0	2 (8.3)
Les Maskoutains	35	1 (2.9)	35	3 (8.6)	4 (11.4)
Sex					
Female	33	1 (3.0)	33	2 (6.1)	4 (12.1)
Male	26	0	26	1 (3.9)	2 (7.7)
Age group^c^					
< 6-month-old	4	1 (25.0)	4	1 (25.0)	1 (25.0)
6-month- to 1-year-old	7	0	7	1 (14.3)	1 (14.3)
>1-year-old	48	0	48	1 (2.1)	4 (8.3)
Sterilised					
Yes	4	0	4	0	1 (25.0)
No	55	1 (1.8)	55	3 (5.5)	5 (9.1)
Body score					
Overweight	10	0	10	0	1 (10.0)
Normal	26	1 (3.9)	26	2 (7.7)	3 (11.5)
Thin	17	0	17	1 (5.9)	2 (11.8)
Emaciated	6	0	6	0	0
Past abortion^d^					
Yes^e^	3	0	3	1 (33.3)	1 (33.3)
No	24	1 (4.2)	24	1 (4.2)	2 (8.3)
Type of farm					
Dairy cattle	43	1 (2.3)	43	2 (4.7)	3 (7.0)
Sheep or goat	16	0	16	1 (6.3)	3 (18.8)
Farm *C. burnetii* (qPCR)^f^					
Positive	8	1 (12.5)	8	1 (12.5)	2 (25.0)
Negative	50	0	50	2 (4.0)	4 (8.0)
Farm *C. burnetii*-status (qPCR and ELISA)^f^					
Positive^g^	23	1 (4.4)	23	2 (8.7)	5 (21.7)*
Negative	35	0	35	1 (2.9)	1 (2.9)
Farm *C. burnetii*-status (qPCR and ELISA)^f^					
Doubtful or positive^h^	25	1 (4.0)	25	2 (8.0)	5 (20.0)*
Negative	33	0	33	1 (3.0)	1 (3.0)
Farm cat origin^f^					
Born at the farm	42	1 (2.4)	42	3 (7.1)	5 (11.9)
Other	14	0	14	0	0
The cat drank raw milk over the past 6 months^f^					
Yes^e^	46	1 (2.2)	46	3 (6.5)	4 (8.7)
No	8	0	8	0	2 (25.0)
The cat hunted a rodent over the past 6 months^f^					
Yes^e^	26	0	26	0	0
No	32	1 (3.1)	32	3 (9.4)	6 (18.9)
The cat hunted a bird over the past 6 months^f^					
Yes^e^	17	0	17	0	0
No	41	1 (2.4)	41	3 (7.3)	6 (14.6)
The cat ate a placenta over the past 6 months^f^					
Yes, cow placenta	7	0	7	0	0
Yes, sheep or goat placenta	5	0	5	0	1 (20.0)
No	46	1 (2.2)	46	3 (6.5)	5 (10.9)
The cat had access to a wooded area over the past 6 months^f^					
Yes	25	0	25	1 (4.0)	2 (8.0)
No	29	1 (3.5)	29	2 (6.9)	4 (13.8)

a Positive to ELISA and/or positive to qPCR.

b Doubtful to ELISA and/or positive to ELISA and/or positive to qPCR.

c Based on the information provided by the owner, available from the questionnaire for 48 cats. For the remaining cats, it was based on the clinical examination.

d Female only; six female cats had missing value.

e Level of the variable identified as a potential risk factor (i.e. higher risk of positivity) and investigated using a Fisher's exact one-sided test. All other variables were tested using two-sided Fisher's exact test.

f For each question, between 1 and 5 cats had a missing value from the questionnaire and/or for the farm status.

g The farm was qPCR-positive and/or ELISA-positive. Doubtful ELISA results were classified as negative.

h The farm was qPCR-positive and/or ELISA-positive and/or ELISA-doubtful.

*P < 0.05, Fisher's exact test for cat results.

Clinical evaluation of the farm cats revealed health issues in 40 cats, including ear mites (*n* = 23 cats), clinical signs suggestive of rhinotracheitis (*n* = 21), cutaneous wound (*n* = 7), presence of fleas (*n* = 6), neurological signs (*n* = 2), dental abscess (*n* = 1) and diarrhoea (*n* = 1). The approximate age of cats was provided by the owner for 48 cats; the median age was 2.3 years, ranging from 3 months to 12 years. Only one of the sampled cats had access to the owner's house over the past 6 months.

**Fig. 1.**
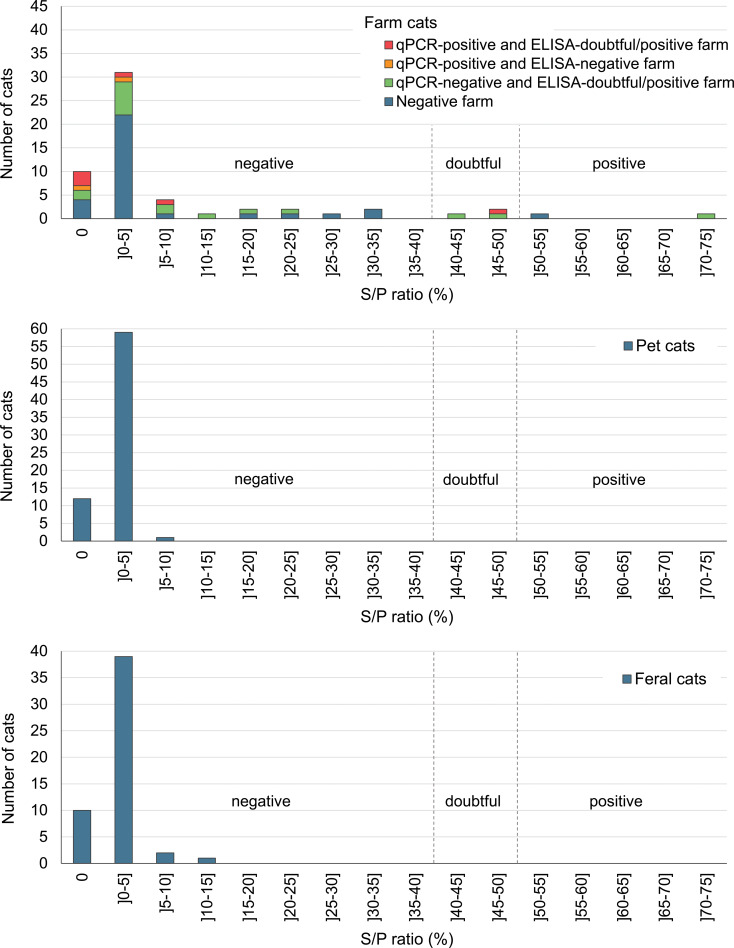
Number of cats over the ELISA S/P ratio (%), for 58 farm cats, 73 pet cats and 52 feral cats. For farm cats, the distribution of cats is shown according to the *C. burnetii*-status of the farm of origin, where a negative farm was ELISA-negative and -qPCR-negative. One farm cat is missing on the graph due to a missing value for farm status. The vertical dashed lines represent the ELISA cut-offs and separate *C. burnetii*-ELISA-negative, -doubtful and -positive cats.

Of the 59 farm cats, only one was *C. burnetii*-qPCR-positive, with an estimated 2.13 × 10^4^ genomic copies per g. This cat was ELISA-negative and was from a dairy cattle farm. Two additional cats were ELISA-positive and three were ELISA-doubtful (Fig. 1). Among these five ELISA-positive or ELISA-doubtful cats, two were from a dairy cattle farm, one from a goat farm and two from a sheep farm. Prevalence estimates are presented in Table 2. Farm positivity to *C. burnetii* was the only statistically significant risk factor identified. Farm cats were more likely to shed the bacteria or have a previous infection detected (i.e. qPCR-positive and/or ELISA-doubtful and/or ELISA-positive) when living on a positive *C. burnetii* farm (PR = 7.6, 95% one-sided CI = (1.33–∞), *P* = 0.03) or on a doubtful or positive farm (PR = 6.6, 95% one-sided CI = (1.15–∞), *P* = 0.04).

**Table 2.** Apparent prevalence of *C. burnetii*-ELISA-doubtful, -ELISA-positive and -qPCR-positive farm cats, pet cats and feral cats in three regions of Quebec, Canada, in 2011–2012

Categories of cats and tests	Number of tested cats	Reacting cats		Prevalence (%)	
		Reaction	Number	Estimate	95% CI
Farm cats					
ELISA	59	Doubtful	3	5.1	1.1–14.2
ELISA	59	Positive	2	3.4	0.4–11.7
qPCR	59	Positive	1	1.7	0.04–9.1
Pet cats					
ELISA	73	Doubtful	0	0	0–4.9
ELISA	73	Positive	0	0	0–4.9
qPCR	69	Positive	0	0	0–5.2
Feral cats					
ELISA	52	Doubtful	0	0	0–6.9
ELISA	52	Positive	0	0	0–6.9
qPCR	52	Positive	0	0	0–6.9

The 73 pet cats were sampled from 29 June 2011 to 15 November 2011, and from 10 July 2012 to 12 September 2012 from all regions. Between 1 and 17 cats were recruited in each of the 7 participating veterinary clinics; in one region, one additional clinic was recruited in 2012 and for both years, the target sample size by clinic was determined according to their available resources. The age of the cat was provided by 67 owners; the median age was 0.8 year, ranging from 12 weeks to 14 years. Most cats (*n* = 62) were presented for an annual examination, vaccination or elective surgery (neutering, declawing); others were presented for various health issues (*n* = 4) or a follow-up examination (*n* = 3). Five cats had a diagnosed chronic health issue (diabetes, urinary infection, chronic renal failure, chronic rhinotracheitis). The questionnaire was completed by all owners except three. The characteristics of the sampled cats are presented in Supplementary Table S1. Four cats were not tested by qPCR due to insufficient faecal material. All tested pet cats were both *C. burnetii*-ELISA- and -qPCR-negative, respectively (Fig. 1 and Table 2).

The 52 feral cats were sampled between 15 June 2011 and 21 September 2011 from a single region, Montreal. Most feral cats sampled were adult intact cats in normal body condition score (Supplementary Table S2). No serum or rectal swab was *C. burnetii*-ELISA- or -qPCR-positive, respectively (Fig. 1 and Table 2).

## Discussion

Our study investigated the prevalence of *C. burnetii* seropositivity and faecal shedding in the farm, pet and feral cats from Quebec, Canada. The presence of the pathogen was previously reported in the three studied regions. In the two rural areas, a concurrent study reported an apparent prevalence of *C. burnetti*-positive 47.3% in 74 dairy cattle herds, 70.8% in 24 sheep flocks and 66.7% in 6 goat herds [23]. Moreover, Q fever cases were regularly reported in the health regions encompassing the study areas over the 10 years preceding our study, with a lower incidence rate in the Montreal region [26].

The rectal swab from one farm cat was qPCR-positive, suggesting that cats could actively transmit the bacteria by faecal excretion. Interestingly, this cat was from a dairy cattle farm in which the bacterium was detected by qPCR in bulk tank milk during the same time period [23]. According to our questionnaire, this cat was 3- to 4-month-old and drank raw milk, a potential source of *C. burnetii* infection in animals and humans [27]. Because this cat's rectal swab was qPCR-positive but its serum was ELISA-negative, it is reasonable to postulate that either the cat was recently infected and had not yet seroconverted or, alternatively, it was a passive *C. burnetii* excretion following ingestion of contaminated raw milk. Indeed, in ruminants, it has been reported that specific antibodies only appear 2 weeks post-infection [28] and shedding of the bacteria can be observed in seronegative animals [29]. Although the possibility of a false-positive qPCR result cannot be ruled out completely, it seems unlikely considering the high analytical specificity reported for a similar assay [25]

When considering both qPCR and ELISA results as indicators of a previous *C. burnetii* infection, the only significant risk factor for farm cat positivity was the detection of a previous infection in the farm ruminant herd. In ruminants, shedding of *C. burnetii* mostly occurs at the time or after parturition [29]. All dairy cattle farms of the study had regular calvings, and all but three of the small ruminant herds had lactating animals at the time of the visit, with the most recent lambing or kidding having occurred on median 14 days before the visit. Moreover, frequent detection of *C. burnetii* in dust samples from ruminant farm buildings had been reported, in agreement with the long-term environmental persistence of the bacteria [30]. Among other potential sources of on-farm exposure, none of the producers witnessed any of the positive cats hunting a rodent in the previous 6 months, suggesting that this source was less likely. However, in part, because cats are often crepuscular or nocturnal, it remains highly possible that such exposure went unnoticed. Overall, in our study, shedding of *C. burnetti* from ruminants represents the most likely source of exposure of farm cats, which could occur through direct contacts, consumption of raw milk or placenta, or environmental exposure.

The association between being a *C. burnetii*-positive farm cat and living on a *C. burnetii*-positive ruminant farm entails that the three doubtful ELISA results obtained in cats were indicative of a previous infection. In this regard, we used cut-offs validated with serum from aborted *C. burnetii*-infected cows [31], as no validated S/P ratio cut-offs relevant to seroprevalence study in cats were available. Compared to use of diagnostic tests in a clinical context, cut-off might need to be lowered in a seroprevalence study to consider the reported decline in specific antibody titers following an acute *C. burnetii* infection [32–34], which has not been evaluated yet in cats. In humans, the half-time of *C. burnetii* antibody decay, as evaluated by the immunofluorescence assay (IFA), was reported to vary from 4 months to 2.5 years depending on the type of antibody and phase [35]. Similarly, in farmed deer, the half-time of antibodies detected by ELISA was estimated to 6 months [34]. In the pets and feral cat populations from our study, which had no evidence of previous infection or exposure to the bacteria, all observed S/P ratios were ≤15%, and 120/124 (97%) of the S/P ratios were ≤5% (Fig. 1). Conversely, the sera from 13/59 (22%) farm cats had S/P ratios >15% and six were from ELISA-positive farms. Taken together, these results suggest that the recommended S/P ratio cut-off for positivity (≥50%) is most likely too high when used to estimate past infection in the context of a seroprevalence study, and doubtful results are most likely indicative of a past infection. According to a seroprevalence study conducted in foxes and cats, which was based on the same ELISA kit (however with some modifications compared to what has been recommended by the manufacturer guidelines), the optimal S/P ratio threshold for positivity was determined to 16.3% based on a bi-model latent class mixture model, which is coherent with our observations [22]. This would imply that our study underestimated *C. burnetii* seroprevalence in farm cats. The validation of the S/P ratio cut-offs in cats, and the assessment of the corresponding ELISA test sensitivity and specificity, would require further investigation.

Farm cats may be involved in the spreading of *C. burnetii* between farms and nearby homes. Many studies have analysed the home range of domestic farm cats and semi-feral farm cats, and most report cats roaming over large areas [36, 37]. In one study, intact male displacement of up to 6.3 km was reported during the mating season [38]. These distances, if applied to farms from the regions sampled, would show a clear overlap of the cat displacement area with nearby farms and/or homes. If a positive farm is a home to a cat shedding the bacteria, this cat becomes a potent and mobile vector for spreading the infection in nearby farms, homes, or even onto other wandering cats. In our study, the only qPCR-positive cat probably had a limited home range due to its young age. Further investigations are needed to assess the risk of faecal shedding in mature, more mobile cats from infected farms. Especially for people who live near farms, these farm kittens or adult cats could represent a source of contamination, since these people may potentially be close to these cats, feeding them or sheltering them occasionally. Investigations of an outbreak that occurred in Nova Scotia revealed another potential source of dissemination: contaminated clothing of workers in contact with parturient cats and newborn and stillborn kittens [15, 17, 39]. Noteworthy, in our study, most farm cats (55/59; 93%) had not been sterilised and 33/59 (56%) were female. Farmers, veterinarians and public health authorities should be aware that exposition to parturient cats, particularly farm cats, is a risk factor for acquisition of Q fever [17, 19]. Neutering of farm cats should also be promoted to reduce their travelling distances and their number on the farm [38].

Our results with pet cats are somewhat different from some of the published literature. Seroprevalence estimates of 14–16% have been reported in Japan, 13% in South Africa, 9% in Korea and 2% in Zimbabwe [9, 40, 41]. However, the cats' origins and ages were not clear, and whether they had been in contact with *C. burnetii* infected farms were not covered. In Quebec, a seroprevalence of 28.1% in 196 cats has been previously reported, but in contrast to our study, sampling was performed following a Q-fever outbreak [20]. Other studies were performed in Canadian provinces east of Quebec. Thus, in Nova Scotia, seroprevalences of 24% to phase II antigens and 6% to phase I antigens were reported in 216 healthy pet cats [42]. In Prince Edward Island, the seropositivity was 7.2% in 97 cats. In New Brunswick, a neighbouring province of Quebec, it was 19.4% among 104 pet cats [43]. It should be noted that the ELISA serological assay used in our work differed from the IFA used in these other Canadian studies, making comparisons difficult [44]. The young age of most pet cats sampled in our study may also have played a significant role in the lower prevalence observed in our study. In fact, as reported for dairy cattle, younger cats might be less likely to be seropositive due to a shorter exposure period [45]. Finally, most pet cats (44/73; 60%) were kept indoors which also limited potential transmission of *C. burnetii* from a prey. This information was usually not provided in other studies making comparisons difficult.

In feral cats, we did not detect any evidence of previous infection or shedding of the bacteria. Previous studies on feral cats showed varying data regarding the presence of *C. burnetii* in feral cat populations. In a study from Japan, 42% of 36 feral cats originating from this country were IFA positive [9]. In a similar study in Colorado, uterine/vaginal samples from 50 shelter cats, which included feral cats, were PCR-negative [10]. Our study is also in agreement with a study conducted in parallel, over the same time period, in the Montreal area, which did not detect *C. burnetii* in cloacal swabs among 187 feral pigeons [46], and with the low incidence rate of human cases of Q fever in this area [26].

Some limitations of our study should be considered when interpreting the results. First, farm cats were not chosen randomly but selected based on approachability, which might have favoured selection of weaker or more sociable cats. However, this selection bias may have a positive side effect of picking cats that are more likely to come in contact with humans, thus making these cats the most desirable subpopulation to study from a public health perspective. Also, we did not reach our targeted sample goal for the farm and feral cat populations, mostly due to a lower number of captured cats than expected during the study period. Moreover, sampling of feral cats was limited to an urban area with no ruminant reservoirs, which overall reduce our ability to detect *C. burnetii* infection if present in feral cats in Quebec. In addition, the qPCR *icd* marker was selected to allow for quantification, but was also reported to have a lower minimal number of genome equivalents detected per reaction compared to the alternative *IS111* marker [25]. Also, the diagnostic sensitivity and specificity on the qPCR assay, as well as the performance of the ELISA assay in cats, were not available from the literature and thus only apparent prevalence results could be reported, and the detection of the *C. burnetii* DNA by qPCR does not determine the bacterial viability. Finally, the statistical power of the study could have been reduced not only by the limited sample size, but also by potential misclassification of exposure to risk factors related to hunting or feeding habits of the farm cats, considering that such exposures were likely under-detected by the cat owners.

In conclusion, we did not detect evidence of active or previous infection with *C. burnetii* in pet cats and feral cats. We were able to demonstrate indications of infection among some farm cats. A positive association was observed between cat and farm status to *C. burnetii.* From a public health perspective, care should be taken when people are in close contact with cats from infected ruminant farms.

## Supplementary material

For supplementary material accompanying this paper visit http://dx.doi.org/10.1017/S0950268821000364. click here to view supplementary material

## Availability of data and materials

The anonymised datasets used during the current study are available from the corresponding author upon reasonable request.
