# Supplementary material for: Prevalence of Coxiella burnetii seropositivity and shedding in farm, pet and feral cats and associated risk factors in farm cats in Quebec, Canada
Source: Epidemiol Infect. 2021 Feb 15;149:e57. doi: 10.1017/S0950268821000364 (PMC8060820; doi:10.1017/S0950268821000364)
Supplement: Supplementary file 1 [file S0950268821000364sup001.pdf]

Epidemiology and Infection

Prevalence of *Coxiella burnetii* seropositivity and shedding in farm, pet and feral cats and associated risk factors in farm cats in Quebec, Canada

J. Cyr, M.-È. Turcotte, A. Desrosiers, D. Bélanger, J. Harel, D. Tremblay, A. Leboeuf, C. A. Gagnon, J.-C. Côté and J. Arsenault

Supplementary material

This supplementary material presents the geographical distribution (Fig. S1) of feral-, farm- and pet cats sampled in Quebec, Canada, for a study on prevalence and risk factors for *Coxiella burnetii* seropositivity and shedding. Farm and pet cats were geo-located at their farm or place of residence, respectively, and feral cats were geolocated at the centroid of their districts of capture, all using GeoPinpoint Suite software version 6.4 (DMTI Spatial Co., Richmond Hill, ON, Canada). Vector files of administrative boundaries were obtained from Statistics Canada (census 2011) and the City of Montreal (Quebec, Canada). The mapping was performed in ArcGIS version 10.5 (Esri Canada, Toronto, ON, Canada).

The characteristics of pet cats (Table S1) and of feral cats (Table S2) are also presented. These characteristics were evaluated using a physical examination and a questionnaire.

Supplementary Fig. S1. Geographical distribution of sampled feral- (n=52), farm- (n=59) and pet cats (n=73) according to their *Coxiella burnetii* qPCR and ELISA status. Top: localisation of the three studied regions within the province of Quebec, Canada, A: Montreal, B: Rimouski-Neigette/La Mitis, C: Les Maskoutains. The exact boundaries of regions are masked to prevent individual identification. The number of feral cats sampled by district is shown using proportional dot symbols.

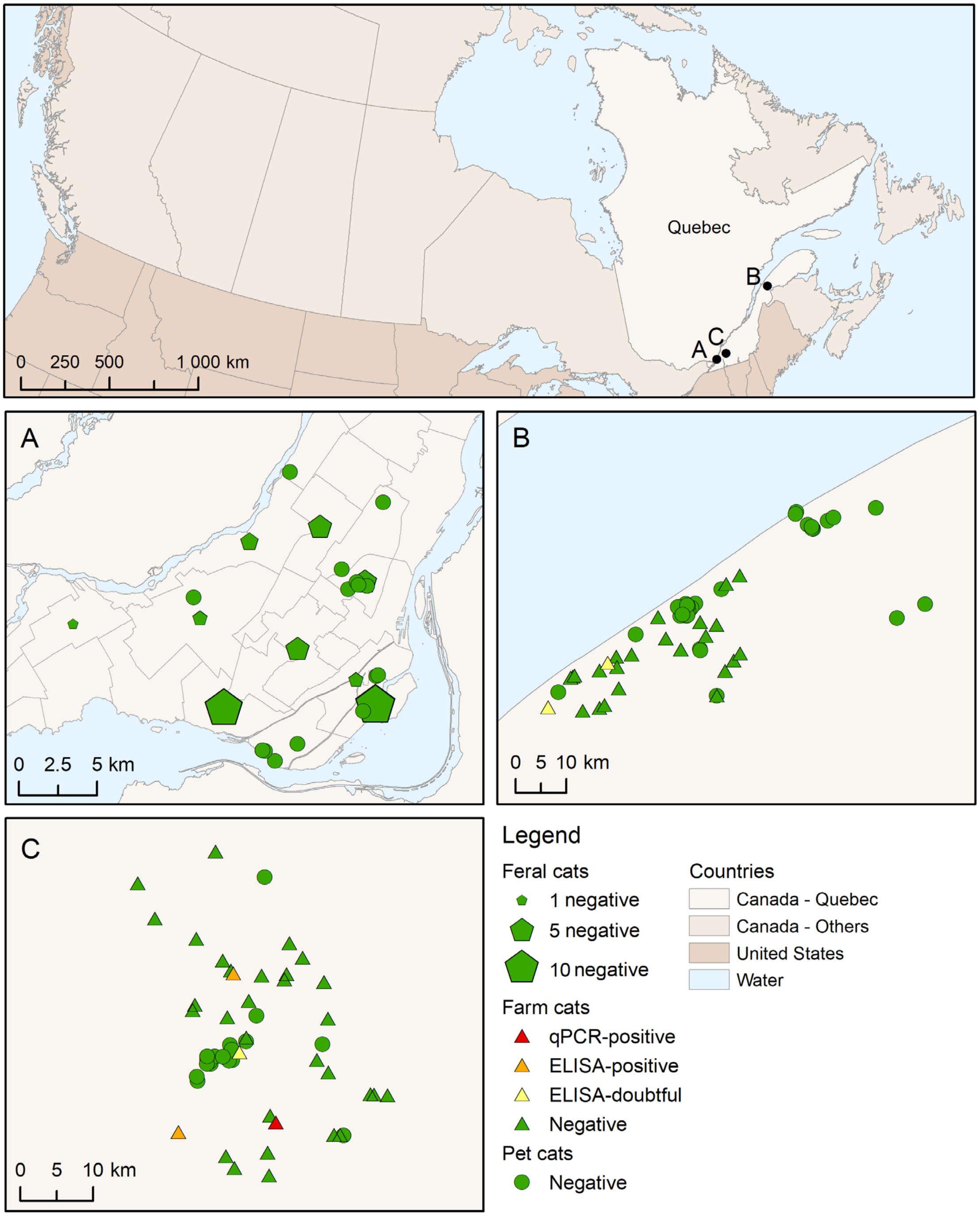

Supplementary Table S1. Characteristics of pet cats sampled in three regions of Quebec, Canada, in 2011-2012

Characteristic	Number of pet cats [a] (n=73)
Region	
Rimouski-Neigette/La Mitis	32
Montreal	19
Les Maskoutains	22
Sex	
Female [b]	34
Male	36
Age group [c]	
< 6-month-old	29
6-month- to 1-year-old	14
> 1-year-old	30
Sterilised	
Yes	56
No	14
Body score	
Overweight	14
Normal	51
Thin	5
Past abortion (female only)	
Yes	1
No	33
Indoor/outdoor cat	
Indoor	44
Indoor and outdoor [d]	26
The cat drank raw milk over the past six months	
Yes	1
No	69
The cat hunted a rodent over the past six months	
Yes	11
No	59
The cat hunted a bird over the past six months	
Yes	5
No	65
The cat ate a placenta over the past six months	
Yes	0
No	70

Characteristic	Number of pet cats [a] (n=73)
The cat had access to a wooded area over the past six months	
Yes	6
No	64
The cat was in contact with farm animals over the past six months	
Yes, including ruminants	1
Yes, other than ruminants	4
No	65
Household member(s) had professional exposure to pet animals over the past six months [e]	
Yes	17
No	53
Household member(s) had professional exposure to farm animals over the past six months [e]	
Yes	3
No	67

[a] 73 pet cats were sampled; data were missing from questionnaire for 3 cats.

[b] None was gestating or lactating; one had a reported previous abortion.

[c] Based on the information provided by the owner available from the questionnaire for 67 cats.

For the remaining cats, it was based on the clinical examination.

[d] Median of 1.5 hours spent outside per day (minimum 2 minutes, maximum 18 hours).

[e] The cat was living in a household in which at least one member had professional contact with pets or farm animals, such as a veterinarian or veterinary technician, or was involved in a training program involving contact with those animals. All reported professional exposure to pets included contact with dogs and/or cats; additional contact with birds and/or iguanas and/or hamsters. Reported professional exposures to farm animals included contact with swine (n=2), cattle (n=2), horses (n=2) and small ruminants (n=1).

Supplementary Table S2. Characteristics of feral cats sampled in Montreal, Quebec, Canada, in 2011

Characteristic	Number of feral cats (n=52) [a]
Sex	
Female [b]	27
Male	23
Age group	
<6-month-old	3
6-month- to 1-year-old	4
>1-year-old	44
Sterilised [b]	
Yes	1
No	49
Body score [b]	
Overweight	2
Normal	42
Thin	7
FIV/FeLV	
Positive [c]	4
Negative	43

[a] A total of 52 cats were sampled. For some variables, data were missing for one to five cats.

[b] Seven females were lactating, and seven others were pregnant.

[c] Positive to at least one of the two viruses.
